# Aircraft noise and self-assessed mental health around a regional urban airport: a population based record linkage study

**DOI:** 10.1186/s12940-018-0418-6

**Published:** 2018-11-11

**Authors:** David M. Wright, Katherine Newell, Aideen Maguire, Dermot O’Reilly

**Affiliations:** 1 Centre for Public Health, Queen’s University Belfast, Institute of Clinical Sciences – Block B, Royal Victoria Hospital, Grosvenor Road, Belfast, BT12 6BA UK; 2 Administrative Data Research Centre-Northern Ireland, Belfast, UK

**Keywords:** Aircraft noise, Mental health, Record linkage

## Abstract

**Background:**

Limited evidence suggests that residential exposure to aircraft noise negatively influences population mental health around large airports, but it is not known whether the same is true for smaller airports. We investigated whether residential exposure to aircraft noise near a regional urban airport was associated with risk of chronic self-assessed mental ill health.

**Methods:**

This is a cross-sectional study of 198,532 people aged 18 years and over living in Belfast, United Kingdom, enumerated at the 2011 Census. Residential exposure to aircraft noise (L_Aeq,16h_) was assessed by linking Census records with modelled noise contours surrounding George Best Belfast City Airport (c.42,000 annual aircraft movements). Associations between noise and mental ill health were estimated using multiple logistic regression adjusting for demographic characteristics, socio-economic status and comorbidity.

**Results:**

Prevalence of self-assessed mental ill health was greater in high noise (≥57 dB) compared to low noise (< 54 dB) areas (12.4% vs. 9.7%). We found no association between aircraft noise and risk of mental ill health after adjustment for socio-economic status (high vs. low noise odds ratio: 1.03 CI: 0.93, 1.14).

**Discussion:**

Associations between aircraft noise and mental health have been reported near large airports at similar average noise levels to those observed here. Our findings indicate that the noise environment around this smaller airport (with fewer flights and no night flights) has little influence on population mental health.

**Electronic supplementary material:**

The online version of this article (10.1186/s12940-018-0418-6) contains supplementary material, which is available to authorized users.

## Introduction

Residential exposure to aircraft noise is increasing rapidly with demand for air transport and has been associated with increased risk of hypertension [1–3], cardiovascular disease and myocardial infarction [4–6], stroke [7] and cardiovascular related mortality [6]. Exposure to aircraft noise has also been associated with reading difficulties and cognitive problems among schoolchildren [8, 9]. Both laboratory and field studies indicate that aircraft noise disrupts sleep, especially towards the end of the night [10–14] and cumulative stress from sleep disruption may explain increased cardiovascular morbidity among those exposed to high noise [15].

Relatively little attention has been given to the long-term influences of aircraft noise on mental health. Poor mental health forms one of the largest components of the global burden of disease [16] so information on the association between aircraft noise and mental health will be crucial when planning future airport expansion. Early studies found area-level associations between noise exposure around major airports (London Heathrow and San Francisco) and hospital admission rates for psychiatric conditions [17–20]. A study of 900 eight to 11 year olds in London revealed weak associations between aircraft noise, hyperactivity and psychological morbidity [9] but a subsequent analysis on a subset of these children found no association between aircraft noise and anxiety, depression or psychological disturbance [21]. Among adults, two major studies of aircraft noise and mental health have been conducted. A cross sectional study of medication use revealed consistent associations between long-term aircraft noise exposure around major airports and elevated anxiolytic use but not antidepressant use across six European countries [22]. This was a branch of the HYENA study of aircraft noise and hypertension [3]. A large longitudinal study (the NORAH study) centred on Frankfurt airport reported a non-linear association between aircraft noise and risk of diagnosed depression among the over 40s [23]. There was a modest increase in depression risk at intermediate levels of noise which disappeared at the highest noise levels, possibly due to health selection. Another Frankfurt based study revealed that aircraft noise was weakly (negatively) associated with self-assessed health-related quality of life and mental health; those most annoyed by aircraft noise or with multiple existing medical conditions reported the greatest decreases in quality of life and mental health with increasing noise [24]. Smaller studies have revealed associations between aircraft noise exposure and increased noise stress and generalised anxiety [25, 26].

In summary, there is weak evidence that aircraft noise negatively influences population mental health around large airports, but little indication whether the same is true for smaller airports. Demand for air transport is expected to increase for both hub and regional airports [27] so it is important to determine the extent to which findings from large airports should inform planning decisions at smaller airports. Large and small airports may produce substantially different noise exposure profiles at similar average noise levels. Average noise exposure measurements may hide differences in maximum noise levels and the number and timing of noise events, parameters that may be independently related to physiological responses, health and wellbeing [11, 13]. For example, the strongest associations between hypertension risk and aircraft noise exposure are with night-time rather than daytime noise levels [1, 2].

In this study we linked two large administrative datasets to investigate whether residential exposure to aircraft noise near a regional urban airport was associated with risk of chronic self-assessed poor mental health. We used data on almost the entire adult population, capturing the full extent of self-assessed poor mental health. Other health conditions moderate the relationship between noise exposure and quality of life [24] so we investigated whether associations between noise and mental health varied with comorbidity.

## Material and methods

### Study location

The study was centred on George Best Belfast City Airport, a single runway airport on a 121 ha site 5 km north east of Belfast (UK) city centre (population 280,000 in 2011). In 2011 there were 41,844 aircraft movements and almost 2,400,000 passengers. Aircraft movements are normally restricted to the hours 06:30 to 21:30. Belfast City Airport was chosen because there is a long running public debate about planned expansion of air traffic in which noise pollution is a central issue and because the flight path crosses an urban area for which information on noise exposure and individual mental health was available.

### Datasets

Estimated residential exposure to aircraft noise was obtained using published noise contours predicted using Integrated Noise Modelling (INM) software [28] for the summer period 16th June to 15th September 2011 [29]. The summer period is frequently used for producing noise contours around UK airports because it is the time of peak aircraft movement due to holiday flights and therefore has the highest noise exposure. The 2011 noise contours were used because these were produced closest to the Census date (27th March 2011). The INM approach evaluates aircraft noise using information on number of aircraft movements on each flight track and noise profiles of each aircraft type. Predictions were validated and adjusted for local conditions using measurements from two noise monitoring terminals within the airport. Contours represented average (A weighted) exposure during the 16-h period 07:00 to 23:00 (L_Aeq 16h_) as this approximates the usual flight operation period. Contours were available at 3 dB intervals in the range 54 to 69 dB (each 3 dB increase represents a doubling of noise energy).

Mental health status and other individual and household characteristics were obtained from the 2011 Northern Ireland Census. The Census contained the question “Do you have any of the following conditions which have lasted, or are expected to last, at least 12 months?” followed by a list of 11 conditions. An affirmative response marked for “an emotional, psychological or mental health condition (such as depression or schizophrenia)” was our measure of self-assessed chronic poor mental health. Because physical conditions are likely to influence mental health, a count of chronic physical conditions (blindness; deafness; mobility difficulties; pain; breathing difficulties; chronic illness) reported for the same question was included as a measure of comorbidity.

Other individual and household characteristics potentially associated with mental health were selected. At the individual level age, sex, ethnicity, religion and marital status were selected. Socio-economic status was characterised by highest educational attainment, household access to cars and housing tenure and value, the latter an indicator of accumulated household wealth [30].

### Data linkage

Individual outcome and covariate data were linked with noise data by overlaying noise contours on household locations; residents were assigned a noise level based on the contour into which the household fell. This was achieved in a geographical information system using point-in-polygon operations to place the georeferenced Census households into noise contour polygons. Following linkage, noise exposure was recategorized into three bands to ensure that population size in each band was sufficient to allow accurate estimation of mental health risk. Bands were: low noise < 54 dB; moderate noise 54–56.9 dB; high noise ≥57 dB. The World Health Organisation sets a threshold of 55 dB above which community noise is likely to cause substantial annoyance [31]. Record linkage and analysis of the de-identified dataset took place within the Administrative Data Research Centre – Northern Ireland. Ethical approval was obtained from the Research Ethics Committee of the School of Medicine, Dentistry and Biomedical Sciences, Queen’s University Belfast.

### Analytical approach

Analysis focused on individuals enumerated at the 2011 Census who were aged 18 years and over and living within the 54 dB Belfast City Airport noise contour plus those outside the contour but resident within Belfast Metropolitan Urban Area (*n* = 198,532). The latter comparator group (i.e. the low noise group) consisted of urban residents not exposed to substantial aircraft noise but nevertheless exposed to higher average noise levels than rural residents, a potential contributory factor towards urban-rural gradients in poor mental health [32].

A series of logistic regression models was fitted to estimate the association between aircraft noise and poor mental health adjusting for the influence of covariates. Robust confidence intervals were estimated to account for household level clustering of poor mental health. An additional model was fitted to investigate potential interactions between comorbidity and aircraft noise and average adjusted predictions (closely related to average marginal effects) were calculated for each interaction level. Analysis was conducted in Stata 13 [33].

A sensitivity analysis was conducted in which all models were refitted, this time excluding those reporting chronic deafness or partial hearing loss (*n* = 13,602), as their mental health is unlikely to have been directly affected by aircraft noise.

## Results

### Aircraft noise and population characteristics

The population characteristics within each noise exposure category are given in Table 1. A greater proportion of individuals in moderate and high noise areas were aged under 45 and had no educational qualifications than in low noise areas. Households in moderate and high noise areas were considerably less likely to have access to cars than those in low noise areas and were more likely to be in rented or lower value accommodation. Prevalence of physical comorbidity was slightly greater in moderate and high noise areas.

**Table 1:** Population characteristics by aircraft noise exposure, Belfast, 2011

		Low noise < 54 dB % (185, 405)	Moderate noise 54–56.9 dB % (8377)	High noise ≥57 dB % (4750)	P (χ^2^ test)
Poor mental health	No	90.3	89.3	87.6	< 0.001
	Yes	9.7	10.7	12.4	
Sex	Male	46.5	47.9	45.8	0.026
	Female	53.5	52.1	54.2	
Age (years)	18–24	15.0	12.5	12.6	< 0.001
	25–34	19.7	27.6	25.8	
	35–44	16.4	17.6	16.9	
	45–54	17.5	14.4	16.5	
	55–64	12.8	10.9	12.1	
	65–74	9.8	9.4	9.4	
	≥75	8.9	7.7	6.8	
Ethnicity	White	97.0	96.7	97.5	< 0.001
	Non-white	3.0	3.3	2.5	
Religion	Protestant	34.7	54.4	64.0	< 0.001
	Catholic	42.7	16.3	7.7	
	Other	22.6	29.3	28.3	
Marital status	Never married	37.3	37.5	35.0	< 0.001
	Married	37.7	28.7	33.6	
	Cohabiting	7.0	13.2	10.7	
	Separated/divorced	10.5	12.7	13.6	
	Widowed	7.5	7.8	7.1	
Educational attainment	No qualifications	30.2	35.0	40.2	< 0.001
	Foundation	17.6	20.5	21.5	
	5+ GCSEs	12.1	11.3	11.5	
	A level	12.5	10.0	9.0	
	Degree	27.6	23.2	17.9	
Physical health conditions	0	67.6	65.7	64.3	< 0.001
	1	15.9	17.1	17.4	
	2	8.1	8.1	8.9	
	3	5.3	5.4	5.9	
	≥ 4	3.2	3.7	3.4	
Household car availability	None	31.5	41.0	38.6	< 0.001
	One	40.5	43.1	43.5	
	Two or more	28.1	15.9	17.9	
Property tenure/capital value	Rented	30.0	44.9	47.2	< 0.001
	Missing	11.8	14.0	9.1	
	<£75 k	8.4	8.2	9.6	
	£75 k-£99 k	13.7	21.1	21.3	
	£100 k–149 k	16.3	9.4	8.9	
	£150 k-£199 k	7.8	1.1	1.5	
	£200 k-£249 k	4.3	0.8	0.8	
	≥£250 k	7.7	0.6	1.5	

### Aircraft noise and mental health

Prevalence of self-assessed poor mental health increased with aircraft noise exposure, a 2.7% increase in high noise compared with low noise areas with moderate noise areas at intermediate risk (Table 1). This translated into a one third increase in the odds of reporting poor mental health in high noise compared with low noise areas (Table 2, Model 1). This pattern persisted following adjustment for age, sex, ethnicity, religion and marital status (Model 2). Following adjustment for education, differences between noise exposure groups in likelihood of poor mental health were attenuated (Model 3: high vs. low noise, odds ratio: 1.13 CI:1.03, 1.25). These differentials disappeared entirely following adjustment for property value and car availability (Model 4). There was a strong exposure-response relationship between the number of physical health conditions and likelihood of poor mental health (Model 5) but no further change in the mental health-noise association following adjustment for comorbidity (Model 5 vs Model 4).

**Table 2:** Associations between residential aircraft noise exposure, individual and household characteristics and risk of self-assessed mental ill health, Belfast, 2011. Odds ratios and robust 95% confidence intervals reported

		Model 1	Model 2	Model 3	Model 4	Model 5
Noise exposure	Low	1.00	1.00	1.00	1.00	1.00
	Moderate	1.12 (1.04, 1.21)	1.11 (1.03, 1.20)	1.04 (0.96, 1.12)	0.92 (0.85, 1.00)	0.92 (0.85, 1.00)
	High	1.33 (1.21, 1.46)	1.32 (1.20, 1.45)	1.13 (1.03, 1.25)	1.04 (0.95, 1.15)	1.03 (0.93, 1.14)
Sex	Male		1.00	1.00	1.00	1.00
	Female		1.29 (1.25, 1.33)	1.34 (1.30, 1.39)	1.32 (1.28, 1.36)	1.34 (1.30, 1.39)
Age (years)	18–24		1.00	1.00	1.00	1.00
	25–34		2.03 (1.89, 2.18)	2.13 (1.99, 2.29)	1.95 (1.82, 2.10)	1.84 (1.72, 1.98)
	35–44		3.95 (3.69, 4.23)	3.51 (3.27, 3.77)	3.45 (3.21, 3.70)	2.85 (2.65, 3.06)
	45–54		4.95 (4.62, 5.31)	3.80 (3.53, 4.09)	3.93 (3.65, 4.23)	2.53 (2.34, 2.73)
	55–64		4.96 (4.60, 5.35)	3.38 (3.12, 3.66)	3.45 (3.18, 3.74)	1.61 (1.48, 1.76)
	65–74		2.56 (2.35, 2.80)	1.50 (1.36, 1.64)	1.48 (1.34, 1.62)	0.55 (0.50, 0.61)
	≥75		1.13 (1.01, 1.26)	0.64 (0.57, 0.72)	0.62 (0.55, 0.70)	0.19 (0.17, 0.22)
Ethnicity	White		1.00	1.00	1.00	1.00
	Non-white		0.38 (0.32, 0.44)	0.40 (0.34, 0.47)	0.32 (0.27, 0.37)	0.38 (0.32, 0.44)
Religion	Protestant		1.00	1.00	1.00	1.00
	Catholic		1.10 (1.06, 1.14)	1.13 (1.09, 1.17)	1.12 (1.08, 1.17)	1.09 (1.05, 1.14)
	Other		0.99 (0.95, (1.04)	1.18 (1.13, 1.24)	1.12 (1.07, 1.17)	1.13 (1.08, 1.19)
Marital status	Married		1.00	1.00	1.00	1.00
	Never married		2.60 (2.48, 2.71)	2.24 (2.14, 2.35)	1.54 (1.46, 1.61)	1.61 (1.53, 1.69)
	Cohabiting		1.28 (1.18, 1.39)	1.21 (1.11, 1.32)	0.92 (0.85, 1.00)	0.98 (0.90, 1.07)
	Separated/divorced		3.72 (3.56, 3.90)	2.93 (2.80, 3.08)	1.96 (1.87, 2.06)	1.87 (1.77, 1.97)
	Widowed		2.39 (2.21, 2.58)	1.87 (1.73, 2.01)	1.39 (1.29, 1.50)	1.26 (1.16, 1.36)
Educational attainment	Degree			1.00	1.00	1.00
	A level			1.61 (1.50, 1.73)	1.41 (1.31, 1.51)	1.31 (1.22, 1.41)
	5+ GCSEs			2.20 (2.06, 2.34)	1.83 (1.72, 1.95)	1.71 (1.60, 1.82)
	Foundation			2.60 (2.46, 2.76)	1.99 (1.88, 2.12)	1.79 (1.68, 1.90)
	No qualifications			4.83 (4.58, 5.10)	3.16 (2.98, 3.35)	2.46 (2.32, 2.61)
Household car availability	Two or more				1.00	1.00
	One				1.56 (1.48, 1.66)	1.47 (1.39, 1.56)
	None				2.31 (2.17, 2.46)	2.17 (2.04, 2.31)
Property tenure/capital value	≥£250 k				1.00	1.00
	£200 k-£249 k				1.29 (1.09, 1.53)	1.23 (1.04, 1.46)
	£150 k-£199 k				1.38 (1.20, 1.60)	1.25 (1.08, 1.45)
	£100 k–149 k				1.52 (1.33, 1.73)	1.30 (1.14, 1.48)
	£75 k-£99 k				1.70 (1.49, 1.93)	1.39 (1.22, 1.58)
	<£75 k				1.66 (1.44, 1.90)	1.33 (1.16, 1.52)
	Rented				2.41 (2.12, 2.74)	1.79 (1.57, 2.03)
	Missing				2.46 (2.16, 2.81)	1.87 (1.64, 2.13)
Physical health conditions	0					1.00
	1					1.98 (1.99, 2.08)
	2					5.32 (5.04, 5.61)
	3					6.98 (6.55, 7.43)
	≥ 4					10.5 (9.69, 11.3)

**Fig. 1:**
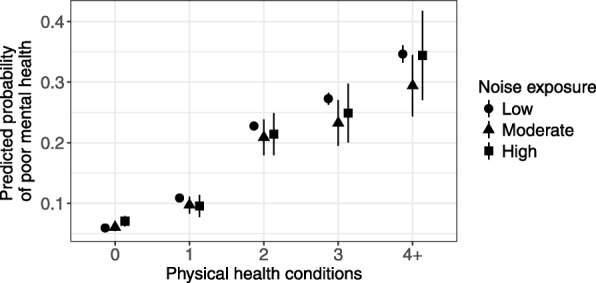
Predicted probability of poor mental health by aircraft noise exposure and number of physical health conditions. Average adjusted predictions and 95% confidence intervals from interaction model between these two variables, adjusting for all other covariates

We found little evidence that comorbidity moderated the association between aircraft noise and mental health. Although there was a statistically significant interaction between comorbidity and noise levels (likelihood ratio test of Model 5 vs. comorbidity by noise interaction model, χ^2^ = 17.19 *P* = 0.028) there was little variation in predicted risk among groups (Fig. 1). Among those with no chronic physical health conditions, those in the high noise area were at marginally increased risk (+ 1%) of poor mental health compared with those in the low noise area but this pattern was absent (i.e. no significant differences between noise exposure groups) among those with physical comorbidity.

### Other variables and mental health

The fully adjusted associations between the other variables and mental health are presented in Table 2, Model 5. Women were more likely to report poor mental health than men and there was a curvilinear relationship between age and likelihood of poor mental health, peaking at 35–44 years of age. Non-Whites were a small minority in the population and were substantially less likely to report poor mental health than Whites. There was subtle variation in risk by religion with Protestants at the lowest risk. Married people and those with higher educational attainment were at reduced risk, as were those with the highest levels of wealth as indicated by house value and car ownership.

### Sensitivity analysis

Exclusion of those reporting chronic deafness made no substantial difference to estimated associations between aircraft noise and poor mental health. All estimates were within the 95% confidence intervals reported for the main analysis (Additional file 1: Table S1).

## Discussion

We found no evidence for an independent association between residential exposure to aircraft noise close to a small urban airport and risk of self-reported poor mental health. Prevalence of poor mental health was greater in moderate and high noise areas (compared with low noise) but these differentials disappeared once measures of socio-economic status (education, housing value/tenure and car availability) were adjusted for. Only for the subgroup of people with no chronic physical conditions was there a negligible increase in risk of poor mental health in the high noise area after adjustment. These findings contrast with two large previous studies: NORAH [23] and HYENA [22] centred on major airports, both of which found associations between mental health (clinician diagnosed depression and anxiolytic use respectively) and average aircraft noise at noise levels similar to those we observed. A possible explanation for this disparity is that measures of self-assessed and clinically diagnosed mental health identify different patient groups. Some with self-assessed poor mental health may have subclinical presentation. Others may report poor mental health in a survey but due to stigma not seek treatment [34, 35]. For example, men are less likely than women to receive inpatient treatment or antidepressant medications at a given level of self-assessed depression [35]. However, our results also contrast with survey-based studies that reported associations between increased aircraft noise exposure and poorer mental health [25, 26, 36]. Therefore, differences in measurement mode alone do not explain the discrepancy in findings between this and previous studies.

An important distinction between the Census and health surveys is that returns are completed at the household level and are visible to other household members, rather than being completed privately by individuals. Evidence suggests that poor mental health was under-reported in the Census relative to uptake of anti-depressants in this population [37], presumably due to stigma associated with declaring poor mental health on a form viewable by others. Therefore, our study is likely to have reported lower prevalence of poor mental health than if a dedicated health survey had been used or diagnosed disease had been the outcome. However, it is likely that the lack of association with aircraft noise would have remained as there is no plausible reason why the proportion of those with a clinical diagnosis willing to report self-assessed poor mental health should vary with noise exposure. Previous work in this population indicates the Census question is a valid measure; health surveys reveal a similar distribution of poor mental health across demographic and socioeconomic groups and Doebler et al. [37] found the same patterns of associations across a broad range of socioeconomic variables regardless of whether Census-based or prescription-based measures of mental health were used. Comprehensive adjustment for socio-economic status strongly influenced the estimated association between aircraft noise and mental health. It was attenuated following adjustment for education and disappeared on inclusion of measures of household wealth. The NORAH [23] and HYENA [22] studies adjusted for socio-economic status using different combinations of variables. In HYENA, only education was adjusted for so reported associations might have altered with inclusion of other measures. This is unlikely with NORAH where education, occupation and social status were used [23]. In NORAH, associations between aircraft noise and mental health persisted following adjustment for socio-economic status, pointing towards systematic differences in population exposure or response to aircraft noise between our study and NORAH rather than differences in adjustment.

Integrated average noise levels were the principle noise exposure measure across all three studies but these do not fully characterise the noise environment around each airport. Other aspects of noise exposure may explain variation in associations between studies. Belfast City Airport had far fewer flights than all but one of the other airports studied in the multi-airport HYENA study with only one tenth of the air traffic of the largest (Frankfurt, London Heathrow and Amsterdam Schiphol). This study [22] found no evidence for variation among airports in the association between average noise and anxiolytic use, despite almost threefold variation in number of flights. Therefore, at high air traffic levels number of flights does not explain variation in mental health beyond the contribution to average noise levels. It is possible that air traffic levels at Belfast City Airport are below a threshold at which noise events are too infrequent to influence mental health regardless of average sound levels.

A more likely explanation centres on timing of noise events. Belfast City Airport does not routinely operate night flights (delayed flights are sometimes allowed to land during the night), in contrast to all but two of the airports featured in the NORAH and HYENA studies (Frankfurt has subsequently introduced such a ban that was associated with changes in self-assessed mental health among those exposed to aircraft noise [36]). Notably, a study of anxiety disorders among residents surrounding an airport of a similar size to Belfast City that has night flights (Cagliari Elmas Airport, Sardinia) revealed associations between mental health and noise exposure [25]. The lack of association between noise and mental health in this study may indicate that night flights have a disproportionate influence on mental health outcomes compared with day flights. The most likely mechanism is sleep disturbance, which is linked with health related quality of life [12, 15, 38] and bidirectionally with mental health outcomes [39] and which may also contribute through stress responses to the negative cardiovascular outcomes reported with high noise exposure [1–3, 15]. Laboratory studies have associated transport noise with reduced subjective sleep quality, daytime sleepiness, sleep fragmentation when event frequencies cross certain thresholds, and subtle changes in other physiological sleep quality measures [10, 11, 40, 41]. Associations differ by traffic type with aircraft and rail noise associated with the strongest reductions in subjective sleep quality but road noise linked with greater physiological disturbance [11]. Increased probability of awakening and reduced subjective sleep quality with aircraft noise exposure have also been observed in field studies, with number of flights and maximum noise levels key predictors of disturbance [14]. Night time maxima were found to be associated with risk of clinical depression in addition to the risk expected based on night time averages [23]. Field studies are few because of considerable technical difficulties accurately measuring sleep in the home [14, 42] and this may explain inconsistencies in findings; some studies find no evidence for associations between noise and sleep quality [43–46]. Another aspect to consider is the way in which mental health may have been influenced by public debate over potential expansion of Belfast City Airport. Pronounced changes in the noise profiles around airports (e.g. opening of a new runway, night-time curfew) have been associated with disproportionately large changes in reported annoyance and population mental health during the transition period [47], likely due to uncertainty about future quality of life. These phenomena are particularly noticeable among those that previously had low noise exposure [36] so this might have led to increased reporting of poor mental health in the low noise exposure group, reducing the observed gradient of poor mental health with noise exposure. No evidence is available to determine whether this mechanism was at work here but the months surrounding the 2011 Census and noise measurements coincided with public scrutiny of a key policy decision to lift a cap on passenger numbers at Belfast City Airport and an enquiry into a proposed runway extension.

The main advantage of using administrative data was availability of individual level records on self-reported mental health and socio-economic status for almost the entire population exposed to aircraft noise around Belfast City Airport. The overall 2011 Census response rate (92%) was considerably higher than for survey-based aircraft noise studies [3, 26], reducing response bias risk. Participants were included from the full adult age range, rather than restricting analysis to middle aged and older people (> 40 for NORAH [23] and ≥ 45 for HYENA [22]). This is important because there are strong trends in poor mental health prevalence with age in this population (Table 2) and excluding those under 40 would exclude a large proportion of those burdened with poor mental health. In this population poor mental health is most commonly found in combination with other conditions [48] and so a strength of our study was that we accounted for the presence of physical health conditions that appear to have a strong influence on mental health.

A limitation of using existing data was that the available noise contours defined exposure categories with relatively narrow ranges, leading us to aggregate across several of the highest contours to ensure that the sample size was sufficient for accurate estimation. This broad ‘high’ noise exposure group encompassed a range of exposures greater than the difference between the low and high groups, potentially masking variation in responses in the noisiest areas. Also, the lower limit of modelled noise exposure (54 dB) was greater than that used in other studies (e.g. NORAH modelled exposure to 40 dB) meaning that we were unable to investigate the noise-mental health association across such a wide range of exposure levels.

Another limitation was that the outcome measure did not distinguish between mental health conditions that may be influenced to varying degrees (or not at all) by aircraft noise. Statistics on prevalence of mental health conditions in Northern Ireland are lacking but based on English statistics [49] it is likely that the majority of individuals reporting poor mental health in this study have either depression, an anxiety disorder or both. Anxiety has been more strongly associated with aircraft noise than depression; two studies found associations with anxiety but not depression [22, 25], the NORAH study [23] found associations with depression using a definition that included mixed anxiety and depressive disorders. Our estimates average across anxiety and depression but we believe a true positive association for either condition should have been detectable given our large cohort (there is no suggestion that aircraft noise protects against any mental condition). Our study had limitations common to environmental noise-health association studies. Noise contours were generated using integrated noise modelling, which does not account for household scale variation in noise due to obstruction by buildings and local topography. This may have led to underestimation of the variation in mental health with noise exposure but we believe any such influence is likely to have been minor; there are few tall buildings and the topography within the noise contours is relatively flat.

At the area level we assumed that aircraft noise during the measurement period represented noise exposure over a longer period, at least in relative terms (i.e. the noisiest areas remained the same over several years). However, residential exposure may not accurately indicate individual noise exposure especially among adults that work elsewhere. This was a cross sectional study so we could not determine the duration of individual exposure to aircraft noise and there may have been some misclassification due to migration. This is likely to have been minor as the population in Northern Ireland is relatively stable with internal migration rates of around 5.8% per year [50]. Therefore, the majority of the cohort was resident in the measured noise exposure bands for several years prior to the study, sufficient time for the most common mental conditions to have developed. Individuals with rarer conditions that may have developed over longer periods or in early life (e.g. schizophrenia) are likely to form a very small proportion of the cohort and consequently have little influence on our estimates. Furthermore, the temporal sequence of noise exposure and deterioration of mental health cannot be determined using this design; individuals may have developed poor mental health before moving to an area exposed to high aircraft noise and others may have moved from the noisiest areas to escape aircraft noise. Poor mental health itself may influence migration, potentially leading to clustering of those with mental health conditions. In this population, long term limiting illness (not divided into mental or physical) was associated with lower migration to less deprived areas [50], possible due to reduced socioeconomic status among those with long term illness. In the absence of longitudinal information on both mental health status and migration we were unable to determine whether this mechanism explains the clustering of poor mental health in more deprived, and in some cases noisier areas. We did not consider other noise sources, potentially obscuring any association between aircraft noise and mental health. Road and rail noise have been associated with increased depression risk, but to a lesser degree than aircraft noise [23]. Studies incorporating multiple noise sources report little difference in estimated aircraft noise–outcome associations for anxiolytic use [22] or cardiovascular outcomes [5, 6] when other noise sources were adjusted for so it is unlikely that such adjustments would substantially alter our findings.

## Conclusions

Using a large linked administrative dataset we found no association between exposure to aircraft noise in the area surrounding a regional urban airport and risk of poor mental health once socio-economic status had been adjusted for. This finding contrasts with two previous studies around larger airports that reported associations at similar average noise levels. Our results may indicate that the noise profile around this airport, with lower air traffic volumes and no night flights has relatively little influence on mental health. Given the rapid global expansion of air travel, further research is needed to determine under which conditions aircraft noise influences population mental health. Future studies should focus on the influence of traffic volumes and night flights as well as average noise exposure. Given the expense of large multi-site surveys, our approach using linked administrative datasets is likely to be cost-effective.

## Additional file

Additional file 1: Associations between residential aircraft noise exposure, individual and household characteristics and risk of self-assessed mental ill health, Belfast, 2011. (DOCX 22 kb)
