# Supplementary material for: Aircraft noise and self-assessed mental health around a regional urban airport: a population based record linkage study
Source: Environ Health. 2018 Nov 11;17:74. doi: 10.1186/s12940-018-0418-6 (PMC6230376; doi:10.1186/s12940-018-0418-6)
Supplement: Supplementary file 1 — Associations between residential aircraft noise exposure, individual and household characteristics and risk of self-assessed mental ill health, Belfast, 2011. (DOCX 22 kb) [file 12940_2018_418_MOESM1_ESM.docx]

# Aircraft noise and self-assessed mental health around a regional urban airport: a population based record linkage study

David M. Wright, Katherine Newell, Aideen Maguire, Dermot O’Reilly

# Supplementary material

Table S1. Associations between residential aircraft noise exposure, individual and household characteristics and risk of self-assessed mental ill health, Belfast, 2011. Cohort excluded those reporting chronic deafness (n = 184,930). Odds ratios and robust 95% confidence intervals reported.

|  |  | Model 6 |
| --- | --- | --- |
| Noise exposure | Low | 1.00 |
|  | Moderate |  |
|  | High |  |
| Sex | Male | 1.00 |
|  | Female | 1.34 (1.29, 1.39) |
| Age (years) | 18-24 | 1.00 |
|  | 25-34 | 1.85 (1.72, 1.99) |
|  | 35-44 | 2.85 (2.65, 3.07) |
|  | 45-54 | 2.56 (2.37, 2.77) |
|  | 55-64 | 1.59 (1.45, 1.74) |
|  | 65-74 | 0.56 (0.50, 0.62) |
|  | ≥75 | 0.20 (0.18, 0.23) |
| Ethnicity | White | 1.00 |
|  | Non-white | 0.36 (0.31, 0.43) |
| Religion | Protestant | 1.00 |
|  | Catholic | 1.07 (1.03, 1.12) |
|  | Other | 1.11 (1.06, 1.17) |
| Marital status | Married | 1.00 |
|  | Never married | 1.63 (1.55, 1.71) |
|  | Cohabiting | 0.99 (0.91, 1.08) |
|  | Separated/divorced | 1.88 (1.78, 1.99) |
|  | Widowed | 1.33 (1.22, 1.46) |
| Educational attainment | Degree | 1.00 |
|  | A level | 1.31 (1.22, 1.42) |
|  | 5+ GCSEs | 1.72 (1.61, 1.84) |
|  | Foundation | 1.80 (1.69, 1.92) |
|  | No qualifications | 2.56 (2.40, 2.72) |
| Household car availability | Two or more | 1.00 |
|  | One | 1.48 (1.40, 1.57) |
|  | None | 2.24 (2.10, 2.39) |
| Property tenure/capital value | ≥£250k | 1.00 |
|  | £200k-£249k | 1.25 (1.05, 1.50) |
|  | £150k-£199k | 1.24 (1.07, 1.45) |
|  | £100k-149k | 1.33 (1.16, 1.52) |
|  | £75k-£99k | 1.38 (1.20, 1.58) |
|  | <£75k | 1.34 (1.16, 1.54) |
|  | Rented | 1.79 (1.56, 2.04) |
|  | Missing | 1.87 (1.63, 2.15) |
| Physical health conditions | 0 | 1.00 |
|  | 1 | 2.04 (1.94, 2.14) |
|  | 2 | 5.47 (5.18, 5.79) |
|  | 3 | 7.11 (6.64, 7.61) |
|  | ≥ 4 | 10.3 (9.33, 11.3) |
